# Changes in the Prevalence of Rheumatic Diseases in Shantou, China, in the Past Three Decades: A COPCORD Study

**DOI:** 10.1371/journal.pone.0138492

**Published:** 2015-09-25

**Authors:** Shao-ying Zeng, Yao Gong, Yu-ping Zhang, Su-biao Chen, Jun-yang Chen, Chu-qing Lin, Jian-hua Peng, Zhi-duo Hou, Jian-qiu Zhong, Hong-jin Liang, Guo-hai Huang, Dan-min Wang, Huai-yuan Lai, Li-ping Li, Qing Yu Zeng

**Affiliations:** 1 The Center for Injury Prevention Research of Shantou University Medical College, Shantou, China; 2 Department of Rheumatology of Shantou University Medical College, Shantou, China; 3 The Third Shantou Municipal Hospital, Shantou, China; 4 The Fourth Shantou Municipal Hospital, Shantou, China; 5 The First Affiliated Hospital of Shantou University Medical College, Shantou, China; Center for Rheumatic Diseases, INDIA

## Abstract

This study aimed to clarify changes in the prevalence of rheumatic diseases in Shantou, China, in the past 3 decades and validate whether stair-climbing is a risk factor for knee pain and knee osteoarthritis (KOA). The World Health Organization-International League Against Rheumatism Community Oriented Program for Control of Rheumatic Diseases (COPCORD) protocol was implemented. In all, 2337 adults living in buildings without elevators and 1719 adults living in buildings with elevators were surveyed. The prevalence of rheumatic pain at any site and in the knee was 15.7% and 10.2%, respectively; both types of pain had a significantly higher incidence in residents of buildings without elevators than was reported by people who lived in buildings with elevators (14.9% vs. 10.6% and 11.32% vs. 8.82%, respectively) (both P < 0.0001). The prevalence of rheumatic pain in the neck, lumbar spine, shoulder, elbow, and foot was 5.6%, 4.5%, 3.1%, 1.4%, and 1.8%, respectively; these findings were similar to the data from the 1987 rural survey, but were somewhat lower than data reported in the urban and suburban surveys of the 1990s, with the exception of neck and lumbar pain. The prevalence of KOA, gout, and fibromyalgia was 7.10%, 1.08%, and 0.07%, respectively, and their prevalence increased significantly compared with those in previous studies from the 20^th^ century. There were no significant differences in the prevalence of rheumatoid arthritis (RA) (0.35%) or ankylosing spondylitis (AS) (0.31%) compared to that reported in prior surveys. The prevalence of KOA was higher in for residents of buildings without elevators than that in those who had access to elevators (16–64 years, 5.89% vs. 3.95%, P = 0.004; 16->85 years, 7.64% vs. 6.26%, P = 0.162). The prevalence of RA and AS remained stable, whereas that of KOA, gout, and fibromyalgia has increased significantly in Shantou, China, during the past 3 decades. Stair-climbing might be an important risk factor for knee pain and KOA.

## Introduction

Since the creation of the International League Against Rheumatism (ILAR), China has cooperated in epidemiological studies of common rheumatic diseases. Research in the field has proceeded gradually in different areas in China, with a focus on identifying the general epidemiology of common rheumatic diseases in China [1]. To determine whether changes in the national economy and people’s living environments and life styles during the past three decades have affected the prevalence of common rheumatic diseases, another epidemiological survey supported by the Asia Pacific League of Associations for Rheumatology (APLAR) was undertaken in 2012 in the Shantou area. This paper reports the results of that survey.

## Materials and Methods

### Study population

Two randomly selected population samples in the Shantou region were surveyed. One population had lived in buildings of 7 to 9 floors without elevators for more than 10 years, and the other had resided in buildings with elevators for more than 10 years. All subjects were 16 years or older. All participants gave their written informed consent after receiving detailed explanations of the study and its potential consequences prior to enrollment. Written informed consent of participants under 18 was given by their parents or caretakers on their behalf. This study was performed with the approval of the Human Ethical Committee of Shantou University Medical College.

### Working team and training

The working team was headed by QYZ and included 6 primary health care workers who were familiar with the local area, 20 senior medical students and resident physicians, 3 rheumatologists, and 2 radiologists. The working team received standard training prior to the survey, including information regarding the survey’s contents, survey procedure, house-to-house survey methods, inquiry techniques, physical examination, daily summary, and data input. A pilot survey was carried out in order to verify the unified criteria and quality of the study. To ensure cooperation among the selected sample population before the survey began, the objective and significance of the study were widely propagandized, and the content and method of the survey were also made known in written form to all participating families.

### Research methods

The protocol of the World Health Organization-International League Against Rheumatism (WHO-ILAR) Community Oriented Program for Control of Rheumatic Diseases (COPCORD) was implemented [2]. The WHO-ILAR COPCORD Core Questionnaire was used by the primary health care workers and the senior medical students for the house-to-house visits during phases I and II; positive respondents identified in phases I and II were interviewed and examined by rheumatologists and senior resident physicians in phase III; relevant laboratory tests and radiographs for subjects suspected of having rheumatic diseases were carried out in phase IV; finally, the rheumatic diseases were characterized and classified, and the data were summarized in the last phase. If a participant was absent or could not be contacted during the survey, another visit was made. All radiographs were independently read by 2 radiologists who were unaware of the clinical data. Finally, 3 rheumatologists made diagnoses according to the examination and test results.

### X-ray examinations

(1) At minimum, frontal radiographs of both hands were taken for the subjects suspected of having rheumatoid arthritis (RA). (2) Frontal radiographs of the pelvis and anteroposterior and lateral radiographs of the lumbar spine were obtained for those suspected of having ankylosing spondylitis (AS). (3) When knee osteoarthritis (KOA) was suspected, anteroposterior and lateral radiographs of the knee joints were taken (4) Finally, in participants who were thought to have gout, anteroposterior and oblique radiographs of the affected foot were obtained to confirm the diagnosis.

### Laboratory examinations

(1) Rheumatoid factor and anti-keratin antibody were tested in subjects suspected of having RA. (2) Those participants in whom ankylosing spondylitis (AS) was suspected underwent HLA-B27 testing. (3) Uric acid levels were tested for those patients thought to suffer from gout.

### Diagnostic criteria

RA was diagnosed according to the American College of Rheumatology (ACR) 2010 criteria [3]. AS was confirmed according to the modified New York Criteria [4]. The ACR 1986 Criteria were used to diagnose KOA [5]. Gout was confirmed according to the ACR 1977 Criteria [6]. Fibromyalgia syndrome was diagnosed using the ACR 1990 Criteria [7].

### Statistical analysis

The prevalence was standardized according to the fifth national census population. The Poisson distribution was used to calculate the 95% confidence intervals (95% CI). Rate comparisons were assessed using the chi-square test. A comparison of the mean values between 2 samples was performed by an independent-samples *t*-test. Logistic regression was used in the risk factors analysis. All statistical analyses were two-tailed and involved the use of the Statistical Package for the Social Sciences (SPSS) version 21.0 (IBM, Armonk, NY, USA). A probability value of P < 0.05 was considered statistically significant.

## Results

A total of 5504 residents lived in the 2 surveyed sites, including 3103 residents in buildings without elevators and 2401 residents in buildings with elevators. The final number of residents actually surveyed was 4056, of which 2337 resided in buildings without elevators and 1719 resided in buildings with elevators. The overall response rate for the questionnaire was 73.7%, and the response rates for those living with and without elevators were 71.6% and 75.3%, respectively. The average age of the 4056 residents surveyed was 48.4 years; there were 1948 men with an average age of 47.2 years and 2108 women with an average age of 49.4 years. The ratio of men to women was 1:1.08. The age and sex of the residents surveyed are shown in Table 1, and their occupations are displayed in Table 2.

**Table 1:** Age, Sex, Rheumatic Pain, Knee Pain, Knee Osteoarthritis, and Gout Distribution of 4056 Respondents in Shantou, 2012.

Age	M		F		RP (%)			KP (%)			KOA (%)			Gout (%)		
(year)	N	%	N	%	M	F	T	M	F	T	M	F	T	M	F	T
16-24	248	###	239	###	3.2	5.4	4.1	2.4	2.9	2.7	0.4	0.8	0.6	0	0	0
25-34	295	###	309	###	4.1	8.7	6.5	2.0	3.9	3.0	0.3	2.6	1.5	0.7	0.3	0.5
35-44	404	###	457	###	11.1	19	15	5.9	13	9.4	1.5	4.8	3.3	2	0	0.9
45-54	404	###	449	###	14.4	28	21	9.9	21	16	5.5	17	12	1.7	0.2	0.9
55-64	310	###	364	###	21.6	37	30	12	29	21	11	26	19	4.2	1.1	2.5
65-74	187	9.7	179	8.5	34.8	53	43	21	29	25	16	34	24	7.5	1.1	4.4
75-84	78	4.1	93	4.4	39.7	53	47	30	42	36	23	32	28	3.9	0	1.8
≥85	22	1.1	18	0.9	31.8	61	###	32	###	###	27	###	38	4.6	0	2.5
Total	###	100	###	100	15.0	26	21	9.4	18	14	6.1	14	10	2.5	0.4	1.4
Sd. R					11.5	20	###	7.0	14	10	4.0	10	7.1	1.8	0.3	1.1

Abbreviations: E = building with elevators; F = female; KOA = knee osteoarthritis; KP = knee pain; M = man; N = number

N-E = building without elevators; RP = rheumatic pain; Sd. R = standardized rate; T = total

**Table 2:** Prevalence Rates and Occupational Distribution of Common Rheumatic Diseases in Shantou, 2012.

		Students	Teachers	IIB	Blue-collar	White-collar	χ^2^	P
		*N* = 206	*N* = 211	*N* = 215	*N* = 873	*N* = 832		
	RP, n (%)	8 (3.88)	59 (27.96)	37 (17.21)	284 (32.53)	177 (21.27)	90.687	<0.001
NE	KP, n (%)	6 (2.91)	36 (17.06)	32 (14.88)	180 (20.62)	109 (13.10)	46.412	<0.001
	KOA, n (%)	1 (0.49)	27 (12.80)	9 (4.19)	151 (17.30)	68 (8.17)	76.576	<0.001
		*N* = 85	*N* = 114	*N* = 294	*N* = 390	*N* = 836		
E	RP, n (%)	3 (3.53)	17 (14.91)	28 (9.52)	87 (22.31)	131 (15.67)	31.207	<0.001
	KP, n (%)	1 (1.18)	14 (12.28)	14 (4.76)	72 (18.46)	96 (11.48)	58.735	<0.001
	KOA, n (%)	−	13 (11.40)	11 (3.74)	55 (14.10)	86 (10.29)	36.758	<0.001
		*N* = 291	*N* = 325	*N* = 509	*N* = 1263	*N* = 1668		
	RP, n (%)	11 (3.78)	76 (23.38)	65 (12.77)	371 (29.37)	308 (18.47)	135.566	<0.001
	KP, n (%)	7 (2.41)	50 (15.38)	46 (9.04)	252 (19.95)	205 (12.29)	105.689	<0.001
Total	KOA, n (%)	1 (0.34)	40 (12.31)	20 (3.93)	206 (16.31)	154 (9.23)	88.213	<0.001
	Gout, n (%)	−	6 (1.85)	12 (2.36)	22 (1.74)	16 (0.96)	12.856	0.009
	RA, n (%)	−	−	2 (0.39)	8 (0.63)	9 (0.54)	2.667	0.595
	AS, n (%)	−	2 (0.62)	1 (0.20)	3 (0.24)	6 (0.36)	2.071	0.719
	FM, n (%)	−	1 (0.31)	−	2 (0.16)	2 (0.12)	1.979	0.758

Abbreviations: AS = ankylosing spondylitis; Blue-collar = blue-collar workers; E = building with elevators; FM = fibromyalgia

IIB = individual industrialists and businessmen; KP = knee pain; KOA = knee osteoarthritis; NE = building without elevators

RA = rheumatoid arthritis; RP = rheumatic pain; White-collar = white-collar workers

Among the 4056 residents surveyed, there were 831, 560, 282, 241, 170, 73, and 111 participants with rheumatic pain, knee pain, neck pain, lumbar spine pain, shoulder pain, elbow pain, and foot pain, respectively, who accounted for 20.5%, 13.8%, 7.0%, 5.9%, 4.2%, 1.8%, and 2.7% of the study population, respectively. The associated prevalence values for each of these types of rheumatic pain were 15.7% (95% CI: 14.7–16.9%), 10.2% (95% CI: 9.5–11.3%), 5.6% (95% CI: 4.9–6.3%), 4.5% (95% CI: 3.9–5.1%), 3.1% (95% CI: 2.6–3.6%), 1.4% (95% CI: 1.0–1.8%) and 1.8% (95% CI: 1.4–2.2%), respectively, after adjusting for age and sex (Table 3).

**Table 3:** Knee Osteoarthritis and Rheumatic Pain at Different Body Sites of the 4056 Respondents in Shantou, 2012.

		KOA			Peripheral				Knee			Neck			Lumbar			Shoulder			Elbow			Foot		
		M	F	T	M	F	T	M	F	T		M	F	T	M	F	T	M	F	T	M	F	T	M	F	T
NE	N	78	178	256	202	363	565	116	246	362		84	131	215	60	124	184	46	87	133	17	48	65	30	33	63
	Pr.%	6.9	14.76	11	17.9	30.1	24.2	10.3	20.4	15.5		7.3	10.9	9.2	5.3	10.3	7.9	4.1	7.2	5.7	1.5	4.0	2.8	2.7	2.7	2.7
	Sd%	4.6	10.8	7.64	13.4^*^ ^&^	23.8^$^	18.5^$^	7.4^*^	15.4^$^	11.3^$^		5.8^*^ ^$^	9.6^$^	7.6^$^	4.3^*^ ^$^	8.0^$^	6.1^$^	2.9^*^	5.5^$^	4.2^$^	1.3^*^ ^&^	2.9^$^	2.1^$^	1.8	1.7	1.7
E	N	40	125	165	90	176	266	69	129	198		20	47	67	18	39	57	17	20	37	3	5	8	35	13	48
	Pr.%	4.9	13.86	9.6	11.0	19.5	15.5	8.4	14.2	11.5		2.4	5.2	3.9	2.2	4.3	3.3	2.1	2.3	2.2	0.4	0.6	0.5	4.3	1.4	2.8
	Sd%	3.22	9.41	6.26	8.9^*^	14.9	11.9	6.5^*^	11.2	8.9		1.8^#^	3.7	2.8	1.4^*^	3.5	2.4	1.5	1.7	1.6	0.2	0.4	0.3	2.9^#^	1.1	2.0
Total	N	118	303	421	292	539	831	185	375	560		104	178	282	78	163	241	63	107	170	10	53	73	65	46	111
	Pr.%	6.06	14.37	10.38	15.0	25.6	20.5	9.4	17.8	13.8		5.4	8.5	7.0	4.0	7.7	5.9	3.2	5.1	4.2	1.0	2.5	1.8	3.3	2.2	2.7
	Sd%	3.97	10.35	7.1	11.5	20.2	15.7	7.0^*^	13.5	10.2	4.3^*^	4.2^*^	7.1	5.6	3.1^*^	6.0	4.5	2.4^#^	3.9	3.1	0.9	1.8	1.4	2.2	1.4	1.8

Abbreviations: E = building with elevators; F = female; M = male; N = number; N-E = building without elevators; T = total; Pr. = prevalence; Sd. R = standardized rate; Comparison with female

* = P < 0.01

# = 0.01 < P < 0.05; Comparison with buildings with elevators

$ = P < 0.01

& = 0.01 < P < 0.05

Among the surveyed population, 421 subjects had KOA, 56 had gout, 19 had RA, 12 had AS, and 5 had fibromyalgia (FM); the associated crude prevalence for each of these conditions was 10.38%, 1.38%, 0.47%, 0.30%, and 0.12%, respectively, and after adjustment, the standardized prevalence was 7.10% (95% CI: 6.31–7.89%), 1.08% (95% CI: 1.02–1.74%), 0.35% (95% CI: 0.17–0.53%), 0.31% (95% CI: 0.14–0.48%), and 0.07% (95% CI: 0–0.15%) (Table 4).

**Table 4:** Distribution of Common Rheumatic Diseases of 4056 Respondents in Shantou, 2012.

	KOA			Gout			RA			AS			FM		
	M	F	T	M	F	T	M	F	T	M	F	T	M	F	T
Number	118	303	421	48	8	56	6	13	19	9	3	12	0	5	5
Prev. %	6.06	14.37	10.38	2.46	0.38	1.38	0.31	0.62	0.47	0.46	0.14	0.30	0	0.24	0.12
Sd. R %	3.97	10.35	7.10	1.82	0.29	1.08	0.23	0.48	0.35	0.51	0.10	0.31	0	0.14	0.07

Abbreviations: AS = ankylosing spondylitis; E = building with elevators; F = female; FM = fibromyalgia; KOA = knee osteoarthritis; M = male; N-E = building without elevators; Prev. = prevalence; RA = rheumatoid arthritis

Sd. R = standardized rate

### The sex, age, housing elevator status, and occupational distribution of subjects with rheumatic pain and knee pain

#### Sex

As shown in Table 3, for subjects living in buildings without elevators, the standardized prevalence of rheumatic pain in women was 23.8% (95% CI: 21.4–26.2%), which was notably higher than the 13.4% (95% CI: 11.4–15.4%) in men (P < 0.001). The standardized prevalence of knee pain in women was 15.4% (95% CI: 11.44–15.68%), which was also notably higher than the 7.4% (95% CI: 5.52–8.71%) in men (P < 0.001); with the exception of the foot, the pain prevalence in other body parts was also higher in women than in men (all P < 0.001). For subjects living in buildings with elevators, the pain prevalence in all parts of the body except the foot was lower than that in subjects living without elevators, regardless of gender (all P < 0.001), and was also significantly lower in men than in women except in the shoulder and elbow (all P < 0.05).

#### Age

As shown in Table 1, the prevalence of both rheumatic pain and knee pain displayed a notably elevated trend after age 35 among the subjects, regardless of whether they were living in buildings with or without elevators and regardless of their gender, but the rising trend was sharper in women than in men.

#### Housing elevator status

As shown in Table 3, the standardized prevalence of rheumatic pain and knee pain was 18.5% (95% CI: 17.4–20.6%) and 11.3% (95% CI: 10.24–12.84%), respectively, in subjects living in houses without elevators and was significantly higher than the 11.9% (95% CI: 9.9–12.9%) and 8.9% (95% CI: 7.55–10.25%) (both P < 0.001), respectively, for subjects living in houses with elevators. The pain prevalence in all parts of the body except the foot was higher in subjects living in houses without elevators than it was in subjects living in houses with elevators (all P < 0.01).

#### Occupation

As shown in Table 2, the prevalence of rheumatic pain was greatest in blue-collar workers (29.37%), followed by teachers (23.38%), white-collar workers (18.47%), individual industrialists and businessmen (12.77%), and finally, students (3.78%). The differences among the groups were statistically significant (χ^2^ = 135.566, P < 0.001). The prevalence of knee pain by occupation had a similar trend, ranging from blue-collar workers (19.95%) to teachers (15.38%), white-collar workers (12.29%), individual industrialists and businessmen (9.04%), and students (2.41%). Again, the differences among the groups were statistically significant (χ^2^ = 88.213, P < 0.001). For participants living in houses without elevators, the prevalence of rheumatic pain was highest in blue-collar workers, followed by teachers, white-collar workers, individual industrialists and businessmen, and finally, students; the differences among the groups were statistically significant (χ^2^ = 90.687, P < 0.001). For subjects who resided in houses with elevators, the prevalence of rheumatic pain was highest in blue-collar workers, followed by white-collar workers, teachers, individual industrialists and businessmen, and finally, students, and the differences among these groups were statistically significant (χ*2* = 31.207, P < 0.001). The prevalence of knee pain in subjects who lived in houses without elevators was highest in blue-collar workers, followed by teachers, individual industrialists and businessmen, white-collar workers, and finally, students, and the differences among the groups were statistically significant (χ*2* = 46.412, P < 0.001). In participants who had regular access to elevators at home, the prevalence of knee pain was highest in blue-collar workers, followed by teachers, white-collar workers, individual industrialists and businessmen, and finally, students, and the differences among the groups were statistically significant (χ*2* = 58.735, P < 0.001).

### The sex, age, housing elevator status, and occupational distribution structure of subjects with common rheumatic diseases

#### Knee osteoarthritis

The prevalence of KOA was more than 2 times greater in women than in men (Table 4). It increased with age; it was less than 2% before age 35, increased notably after age 45, reached 24.3% at age 65–74, and was 37.5% at age 85 or older (Table 1). As shown in Table 3, the total standardized prevalence of KOA was higher in subjects living without elevators than in subjects with elevators (7.64% vs. 6.26%, P = 0.162) and was significantly higher in subjects aged 16–64 who resided in buildings without elevators than it was for those with access to elevators (5.89% vs. 3.95%, P = 0.004). As shown in Table 2, the prevalence of KOA was highest in blue-collar workers, followed by teachers, white-collar workers, individual industrialists and businessmen, and finally, students. Differences among the groups were statistically significant (χ^2^ = 105.689, P < 0.001).

#### Gout

The prevalence of gout was 6 times higher in men than in women (1.82% vs. 0.29%) (Table 4). The prevalence of this condition also increased with age and reached a peak at ages 65–74; except for one female patient who was younger than 35, no men under 30 or women under 45 had gout (Table 1). The prevalence of gout was highest in individual industrialists and businessmen, followed by teachers, blue-collar workers, and white-collar workers; there were no students with gout (Table 2). Differences among the groups were statistically significant (χ^2^ = 12.856, P = 0.009).

#### Rheumatoid arthritis and ankylosing spondylitis

The prevalence of RA was 0.35% (95% CI: 0.17–0.53%), and was more than 2 times higher in women than in men (0.48% vs. 0.23%, 95% CI: 0.18–0.78% and 0.02–0.44%). The prevalence of AS was 0.31% (95% CI: 0.14–0.48%), and it was 5 times higher in men than in women (0.51% vs. 0.10%, 95% CI: 0.19–0.83% and 0–0.23%). These 2 rheumatic diseases showed no evident relationship with housing elevator status or occupation (Table 2, Table 4).

#### Fibromyalgia

All 5 subjects with fibromyalgia were female. The prevalence was 5/4056 (Table 4). The ages of those with fibromyalgia ranged from 45–64. Among the 5 subjects, 2 were blue-collar workers, 2 were white-collar workers, and 1 was a teacher (Table 2). All the above subjects reported being under psychological stress.

### Changes in the prevalence of common rheumatic diseases in Shantou in the past 3 decades, and comparison of these changes with those in other major cities (Table 5) and countries

#### Rheumatic pain

Compared with the rural survey results of 1987 [2], in the current survey, there was a lower prevalence of lumbar pain (4.5% vs. 13.0%, P < 0.001), a similar prevalence of shoulder, elbow, and foot pain, and a higher prevalence for all other body parts (15.7% vs. 11.6%, P < 0.001), knee pain (10.2% vs. 2.6%, P < 0.001), and neck pain (5.6% vs. 2.0%, p<0.001). Compared with the town survey results of 1992 [8], except for a higher prevalence in all parts of the body and a lower prevalence both in lumbar pain and neck pain, the prevalence results in all other parts of the 2 surveys were similar. Compared with the urban survey results of 1995 [9, 10] and the rural survey results of 1999 [11–13], the prevalence of rheumatic pain in all parts of the body was lower (15.7% vs. 18.1% and 19.8%; P = 0.018 and P < 0.001, respectively) in particular for subjects living in buildings with elevators. The prevalence of rheumatic pain in this survey was also lower than the rate previously reported in Taiwan [14], Shanghai in both 1992 and 1998 [15], Taiyuan [16], and Beijing [2] (15.7.0% vs. 23.0%, 24.3%, 21.2%, 25.4%, and 40.3%, respectively) (all P < 0.001).

**Table 5:** Prevalence Rates (%) of Rheumatic Pain and Common Rheumatic Diseases in Different Areas and During Different Time Periods.

	Shantou (23)^#^								Taiwan (25)^#^					Shanghai (32)^#^					Taiyuan (37)^#^	Beijing (40)^#^			
	**1984**	**1987**	**1992**	**1995**	**1999**	**2012 (Urban)**			**1994**			**-1996**	**-2008**	**1992**	**1997**	**1998**	**2001**	**2002**	**2004**	**1987**	**1994**	**2005**	**2011**
Ref. N	**31**	**2**	**8, 32**	**9, 10**	**13**	**current**			**14**			**19**		**1、15**	**20**	**21**	**17**	**22**	**16**	**1、2**	**18**	**24**	**23**
Location	Ur-Ru	Rur	Urb	Urb	Sub	N-E*	E*	Total	Urb	Sub	Rur	NAHSIT		Urb	Urb	Urb	Urb	U+R	Urb	Rur	Rur	Urb	Rur
Number	10647	5057	1722	2040	1818	2337	1719	4056	3000	3000	2998	2979	1661	2010	2037	6584	2093	7575	3915	4192	2063	1997	10056
M:F	1:0.9	1:1.10	1:0.89	1:1.07	1:1.11	1:1.06	1:1.10	1:1.08	1:0.96	1:1.03	1:0.93	1:1.20	1:1.05	1:1.20	1:1.23	1:1.05	1:1.3	1:1.05	1:1.11	1:1.01		1:0.6	1:1.0
Age	≥16	≥20	≥20	≥16	≥16	≥16	≥16	≥16	≥20	≥20	≥20	≥19		≥16	≥15	≥16	≥40	≥15	≥16	≥	≥16	≥20	≥16
Peripheral		11.6^$^	12.5	18.1^&^	19.8^&^	18.5	11.9	15.7	26.3	18.4	24.3			24.3		21.2			25.4	40.3			
Knee		2.6^&^	7.5	7.5	12.5^&^	11.32	8.82	10.20						10.9		7.0	9.9		9.8	30			
Lumbar		13.0^&^	11.2^&^	11.5^&^	10.8^&^	6.1	2.4	4.5						5.8		5.6			8.5	35			
Shoulder		2.0		5.3^&^		4.2	1.6	3.1						4.8		4.7			-	5			
Neck		2.0^&^	8.9^&^	4.6	7.1^&^	7.6	2.8	5.6						3.5		2.4			5.0	5			
Elbow		1.4	0.9			2.1	0.3	1.4												4.4			
Foot		2.0^$^	2.3			1.7	2.0	1.8												1.5			
KOA			1.3	3.2		7.64	6.26	7.10								4.1	7.2	4.18	7.57		9.6		
Gout			0.17	0.15	0.26	1.15	0.96	1.08	0.67	0.67	0.16	3.4	5.2	0.20	0.34	0.22		0.28				1.0	0.09
RA		0.32		0.2	0.26	0.35	0.35	0.35	0.93	0.78	0.26			0.20		0.28		0.52	0.28	0.34			0.28
AS	0.20	0.26		0.2	0.3	0.31	0.31	0.31	0.4	0.19	0.54			0.20		0.11		0.28	0.20	0.26			
FM						0.10	0.03	0.07															

Abbreviations: AS = ankylosing spondylitis; E = building with elevators; F = female; FM = fibromyalgia; KP = knee pain; KOA = knee osteoarthritis; M = male; N-E = building without elevators; RA = rheumatoid arthritis; Ref. N = reference number; Rur = rural; Sub = suburban; Urb = urban

^#^ The figure in parentheses is the degree of latitude; Comparison with Shantou of 2012

^$^0.01 < p < 0.05

^&^p < 0.01.

#### Knee osteoarthritis

There was an increased prevalence of KOA in Shantou compared with that in 1992 and 1995 (7.10% vs. 1.3% and 3.2%, respectively) (both P < 0.001) [8, 9], and the increase was more evident in subjects who lived in buildings without elevators than in those who had access to elevators (7.64% vs. 6.26%). The prevalence of KOA in this study was similar to those reported in Taiyuan (7.57%) [16] and Shanghai (7.2%) [17], but it was still lower than that in Beijing (9.6%, P = 0.064) [18].

#### Gout

The prevalence of gout in Shantou was higher in the current study than it was in those reported in 1992, 1995, and 1999 (1.08% vs. 0.17%, 0.15%, and 0.26%, respectively; all P < 0.01) [11]. The prevalence was also higher than that of rural, suburban, and urban Taiwan in 1994 (1.08% vs. 0.16%, 0.67%, and 0.67%; P < 0.001, P = 0.067, and P = 0.067, respectively) [14), and higher than that reported in Shanghai in 1992, 1997, 1998, and 2002 [15, 20–22] and in Beijing in 2011 [23] (1.08% vs. 0.2%, 0.34%, 0.22%, 0.28%, and 0.09%, respectively; (all P < 0.01). However, the prevalence of gout in the current survey was lower than that of the NAHSIT (the two Nutrition and Health Surveys in Taiwan) survey results in 1993–1996 and 2005–2008 (1.08% vs. 3.4%, 5.2%, respectively; both P < 0.001) [19] and was similar to that of the cadres examination in Beijing in 2005 (1.0%) [24].

#### Fibromyalgia

The prevalence of fibromyalgia in the current study was higher than that of the former native survey results in 2004 [25] and the Taiyuan survey result in 2007 [16] (5/4056 vs. 2/2350 and 1/3915), but it was still lower than that reported in other countries, including the United States (US) [26], Japan [27], India [28], Bangladesh [29], and Mexico [30].

#### Rheumatoid arthritis and ankylosing spondylitis

The prevalence rates of RA and AS showed no evident changes over time, and they were similar to those in other civil survey results. The prevalence of RA was similar to that in India [31], but was lower than that in Mexico [30] and Caucasians, while the prevalence of AS was similar to that found in Western countries [1], but lower than that in India [31].

### Factors associated with rheumatic diseases

Logistic regression was adopted to analyze the effects of sex, age, occupation, and housing elevator status on rheumatic diseases. The result showed that age, sex, stair-climbing, and occupation were all risk factors for knee pain and knee osteoarthritis, and that sex and age were risk factors for gout (Table 6).

**Table 6:** Logistic Regression Analysis of Knee Pain, Knee Osteoarthritis, and Gout with Sex, Age, Access to Elevator, and Occupation.

	Regression coefficient	Wald	P Value	OR Value(95% CI)
Knee pain				
Sex	0.920	77.623	< 0.001	2.509 (2.045, 3.079)
Age	0.057	297.549	< 0.001	1.059 (1.052, 1.065)
Elevator	0.081	22.252	< 0.001	1.084 (1.049, 1.122)
Occupation	0.141	15.838	< 0.001	1.152 (1.074, 1.235)
Knee Osteoarthritis				
Sex	1.116	83.531	< 0.001	3.051 (2.402, 3.876)
Age	0.066	294.007	< 0.001	1.068 (1.060, 1.076)
Elevator	0.053	7.066	0.008	1.054 (1.014, 1.096)
Occupation	0.171	17.945	< 0.001	1.187 (1.096, 1.285)
Gout				
Sex	-2.016	29.845	< 0.001	0.133 (0.065, 0.275)
Age	0.036	19.646	< 0.001	1.037 (1.020, 1.053)
Elevator	-0.046	0.796	0.372	0.955 (0.862, 1.057)
Occupation	0.142	1.917	0.166	1.152 (0.943, 1.408)

Abbreviations: CI = confidence interval; OR = odds ratio.

## Discussion

COPCORD was introduced by the WHO and ILAR last century to help developing countries control rheumatism. It proposed 3 stages: the first is an epidemiological, community-based survey of rheumatic diseases; the second includes treatment and health education for rheumatic diseases; and the third involves validation of the risk factors for rheumatism, both environmental and genetic, in order to prevent or decrease rheumatism [32].

By participating in the ILAR-China epidemiological survey of Chinese rheumatism from the early 1980s to the late 1990s, the Rheumatology Department of Shantou University Medical College carried out successive surveys among 5 cohorts, including a total of 23,867 residents over 16 years of age [8–13, 33, 34], and found that there was a rising trend in the prevalence of rheumatic pain and an increase in the prevalence of gout in the Shantou area. The change in the prevalence of these common rheumatic diseases was considered to be related with a change in people’s socioeconomic status and living habits, and stair-climbing was considered to be a risk factor for knee pain and knee osteoarthritis.

All questionnaires used in the aforementioned surveys were identical to the WHO-ILAR COPCORD Core Questionnaire [35]; the only difference was that the subjects were examined soon after the questionnaires, whereas in the WHO-ILAR COPCORD Core survey, medical examinations were carried out within 3 days after the participants responded to the questionnaires [13]. All questionnaires used in the above surveys were translated from Chinese to English and then from English back to Chinese, and they were validated by clinical verification and small-scale investigations [36–38]. Moreover, there was a stable working team for the surveys in which members including the research director, rheumatologists, radiologists, and even the supervisors from the Chinese Rheumatism Association, APLAR, and ILAR were unchanged.

The current survey was consistent with the previously mentioned COPCRD surveys. All these consistent surveys were conducted in Shantou area, where the overwhelming majority of the population has always remained Han people due to its culture and situation. Moreover, the working team and the methodology remained unchanged, greatly decreasing the potential for errors in the surveys and therefore ensuring comparable results over the past 3 decades.

With land prices soaring due to urbanization and the expansion of city scales in the past 3 decades, buildings without elevators have risen higher and higher, while buildings with elevators sprang up around the year 2000 in Shantou and have become as common as buildings without elevators. In contrast, almost all subjects in the previous COPCORD surveys conducted in Shantou from 1987 to 1999 lived in buildings without elevators, so the current survey was conducted in two randomly selected population samples with and without elevators separately to further ensure comparable results.

### Rheumatic pain

There have been 5 rheumatic epidemiological surveys on rheumatic pain since the 1980s in Shantou, and the prevalence of rheumatic pain has showed an upward trend from 1987–1999 but a decrease from 1999 until our survey was carried out. These results might be related to people’s strong work ethic and the fierce competition in the workforce since the 1980s, as well as the corresponding increase in the social economy and increasingly higher living standards, lower labor intensity, and the development of health services since the turn of the century.

### Knee pain and knee osteoarthritis

The survey showed that the prevalence of knee pain and KOA had increased despite the overall declining prevalence of rheumatic pain, and it suggested that knee pain and KOA were not only related with stair-climbing but also with occupations.

Our 1995 survey showed that the prevalence of knee pain, lumbar pain, and degenerative changes in residents living on the 4^th^ to 5^th^ floors of buildings without elevators was twice that of residents living in single-story houses [10], but we could not verify the relationship between stair-climbing and knee pain or KOA in a later study in Taiyuan [39]. The results of the current survey showed that both the prevalence of knee pain and KOA were higher in residents living in buildings without elevators than in buildings with elevators, suggesting that the higher prevalence of knee pain and KOA might be related to stair-climbing. However, the different effects of stair-climbing on different age groups will require further clarification.

Regarding the 2006 Taiyuan report [39], the relationships between stair-climbing and the prevalence of knee pain or KOA were drawn from comparisons of the prevalence rates in residents living on different floors in buildings without elevators, which had some limitations: one limitation was the lack of a comparison with residents living in buildings with elevators or in single-story houses, and the other was that there were too many confounding factors, such as the residents’ distribution on different floors being related to their social status for buildings owned by an employer, or that older and weaker people or those with knee trouble tended to live on the lower floors.

With urbanization and the expansion of cities over the past 3 decades, land prices have soared, resulting in little to no construction of single-story houses except for a few luxury villas in both urban and more rural settings; in fact, even blocks of buildings under 4–5 stories without elevators are scarce. Therefore, it is difficult to carry out a cohort study between single-story houses and buildings without elevators. The comparison in this study was between residents living in buildings with and those without elevators, which was a rational cohort to study the association between stair-climbing and knee pain or KOA. The buildings in the 2 selected survey sites were completed at almost the same time 10 years ago; the houses were bought individually, and the residents moved into their residences at almost the same time. Therefore, there were few possible confounding factors in the survey. The survey results showed no apparent differences in the prevalence of KOA in residents younger than 40 years of age, regardless of whether their residence had elevators. The difference in KOA prevalence appeared after 45 years of age, which was in accordance with the development of KOA and previous epidemiological survey results [40], suggesting the reliability of the survey results.

The reason for the increase in the prevalence of knee pain and KOA despite the decreasing overall prevalence of rheumatic pain is worthy of attention. The increased prevalence of knee pain and KOA may have been affected by the general prolongation of the life span due to economic development during the past three decades, which has resulted in a larger population of older people, who typically report a higher prevalence of knee pain and KOA.

The survey results suggested that knee pain and KOA were not only associated with stair-climbing but also with a person’s occupation. When adjusted for age and sex, both the prevalence of knee pain and KOA were higher in residents living in buildings without elevators than in buildings with elevators. However, people who already had knee pain or KOA likely had a tendency to move to houses with elevators. At the same time, being sedentary may have falsely “improved” the pain, so it was thought that the prevalence rates of knee pain and KOA would be higher in subjects who chose to live in buildings with elevators than in those who moved into residences without elevators 10 years ago. However, the survey results 10 years later were contrary, which further verifies the effect of stair-climbing on knee pain and KOA, but no data regarding the prevalence of knee pain or KOA were obtained 10 years ago. Other factors, such as socioeconomic status, body mass index, waist circumference, comorbidities, education level, and smoking history, might also have contributed to the development of knee pain or KOA. Therefore, further study will be required to determine the role of these factors and explore their interactions.

### Gout

The prevalence of gout has shown a rising trend in Shantou since the 1990s [11], and it has also risen in both mainland China [41] and Taiwan [19]. Our survey results showed that the prevalence of gout in Shantou was currently significantly higher than that at the end of the last century, which is undoubtedly related to the rapid development of national economics, greatly improved living standards, and changed lifestyles in the past 3 decades in mainland China; however, the prevalence of gout is still lower than that reported by Western countries in the 1990s [42]. So far, survey results on gout in various areas of China or among various cohorts in the same area have differed. Therefore, it has been suggested that both genetics and lifestyle may influence this disease.

### Fibromyalgia

With the exception of the surveys conducted in Taiyuan and Shantou by our research center, no epidemiological data on FM have been obtained in China [16, 25]. It is noteworthy that the prevalence of FM in this survey was higher compared to the survey results from 2004 and 2007 (5/4056 vs. 2/2350 and 1/3915). The 5 women with FM in this survey were approximately 50 years old, and they all reported experiencing mental or psychological pressure from their children’s employment or other domestic problems, which suggests the importance of considering people’s mental and psychological pressure during the rapid development of social economics, particularly in those vulnerable to such pressures. Although the prevalence of FM was higher in our study compared with that in the previous surveys, it was still lower than that reported in western countries, which may be related to the different paces of life, working pressures, or genetic factors in western countries. Further studies are needed to investigate this relationship.

In conclusion, in the past 3 decades, the prevalence of RA and AS remained stable, whereas the prevalence of KOA, gout, and FM increased notably; these changes occurred alongside major changes in the social economic status and overall lifestyle in China. Age, sex, stair-climbing, and occupation were all risk factors for knee pain and knee osteoarthritis, and sex and age were also risk factors for gout. More risk factors should be investigated in future studies.

## Supporting Information

S1 Table The Age, Sex, and Rheumatic Pain Distribution of the 4056 Respondents in Shantou, 2012 (2–1) (2–2) (DOC)

S2 Table Prevalence Rates and Occupational Distribution of Common Rheumatic Diseases in Shantou, 2012 (2–1) (2–2) (DOC)

S3 Table Age, Sex, and Housing Distribution of Common Rheumatic Diseases in Shantou, 2012 (DOC)
