# Supplementary material for: Changes in the Prevalence of Rheumatic Diseases in Shantou, China, in the Past Three Decades: A COPCORD Study
Source: PLoS One. 2015 Sep 25;10(9):e0138492. doi: 10.1371/journal.pone.0138492 (PMC4583180; doi:10.1371/journal.pone.0138492)
Supplement: S1 Table — (DOC) [file pone.0138492.s001.doc]

**S1Table.** The Age, Sex, and Rheumatic Pain Distribution of the 4056 Respondents in

Shantou, 2012（2-1）

|  | Age | Male | |  | Female | |  | Total | |  | Peripheral (％) | | |  | Knee (％) | | |  |
| --- | --- | --- | --- | --- | --- | --- | --- | --- | --- | --- | --- | --- | --- | --- | --- | --- | --- | --- |
|  | （yrs） | n | % |  | n | % |  | n | % |  | M | F | Total |  | M | F | Total |  |
| NE | 16-24 | 136 | 12 |  | 138 | 11.4 |  | 274 | 11.7 |  | 2.9 | 2.9 | 2.9 |  | 2.9 | 1.5 | 2.2 |  |
|  | 25-34 | 194 | 17.2 |  | 196 | 16.3 |  | 390 | 16.7 |  | 3.6 | 11.7 | 7.7 |  | 1.5 | 3.6 | 2.6 |  |
|  | 35-44 | 217 | 19.2 |  | 239 | 19.8 |  | 456 | 19.5 |  | 13.4 | 23.0 | 18.4 |  | 5.5 | 14.2 | 10.1 |  |
|  | 45-54 | 220 | 19.5 |  | 262 | 21.7 |  | 482 | 20.6 |  | 17.7 | 38.2 | 28.8 |  | 11.4 | 26.0 | 19.3 |  |
|  | 55-64 | 196 | 17.3 |  | 224 | 18.6 |  | 420 | 18 |  | 27.6 | 46.4 | 37.6 |  | 14.3 | 34.8 | 25.2 |  |
|  | 65-74 | 116 | 10.3 |  | 82 | 6.8 |  | 198 | 8.5 |  | 38.8 | 54.9 | 45.5 |  | 22.4 | 37.8 | 28.8 |  |
|  | 75-84 | 39 | 3.4 |  | 52 | 4.3 |  | 91 | 3.9 |  | 51.3 | 44.2 | 47.3 |  | 30.8 | 40.4 | 36.3 |  |
|  | ≥85 | 13 | 1.1 |  | 13 | 1.1 |  | 26 | 1.1 |  | 38.5 | 69.2 | 53.9 |  | 38.5 | 53.8 | 46.2 |  |
|  | Total | 1131 | 100 |  | 1206 | 100 |  | 2337 | 100 |  | 17.9 | 30.1 | 24.2 |  | 10.3 | 20.4 | 15.5 |  |
|  | Standardized Rate |  |  |  |  |  |  |  |  |  | 13.4*^&^ | 23.8^$^ | 19.0^$^ |  | 7.4* | 15.4^$^ | 11.5^&^ |  |
| E | 16-24 | 112 | 13.7 |  | 101 | 11.2 |  | 213 | 12.4 |  | 3.6 | 8.9 | 6.1 |  | 1.8 | 5.0 | 3.3 |  |
|  | 25-34 | 101 | 12.4 |  | 113 | 12.5 |  | 214 | 12.4 |  | 5.0 | 3.5 | 4.2 |  | 3.0 | 4.4 | 3.7 |  |
|  | 35-44 | 187 | 22.9 |  | 218 | 24.2 |  | 405 | 23.6 |  | 8.6 | 13.8 | 11.4 |  | 6.4 | 10.6 | 8.6 |  |
|  | 45-54 | 184 | 22.5 |  | 187 | 20.7 |  | 371 | 21.6 |  | 10.3 | 12.8 | 11.6 |  | 8.2 | 15.0 | 11.6 |  |
|  | 55-64 | 114 | 14 |  | 140 | 15.5 |  | 254 | 14.8 |  | 11.4 | 22.9 | 17.7 |  | 8.8 | 18.6 | 14.2 |  |
|  | 65-74 | 71 | 8.7 |  | 97 | 10.8 |  | 168 | 9.8 |  | 28.2 | 50.5 | 41.1 |  | 19.7 | 21.6 | 20.8 |  |
|  | 75-84 | 39 | 4.7 |  | 41 | 4.5 |  | 80 | 4.6 |  | 28.2 | 63.4 | 46.3 |  | 28.2 | 43.9 | 36.3 |  |
|  | ≥85 | 9 | 1.1 |  | 5 | 0.6 |  | 14 | 0.8 |  | 22.2 | 40.0 | 28.6 |  | 22.2 | 40.0 | 28.6 |  |
|  | Total | 817 | 100 |  | 902 | 100 |  | 1719 | 100 |  | 11.0 | 19.5 | 15.5 |  | 8.4 | 14.2 | 11.5 |  |
|  | Standardize Rate |  |  |  |  |  |  |  |  |  | 8.9* | 14.9 | 11.4 |  | 6.5* | 11.2 | 8.9 |  |
| Total | 16-24 | 248 | 12.7 |  | 239 | 11.3 |  | 487 | 12 |  | 3.2 | 5.4 | 4.1 |  | 2.4 | 2.9 | 2.7 |  |
|  | 25-34 | 295 | 15.1 |  | 309 | 14.7 |  | 604 | 14.9 |  | 4.1 | 8.7 | 6.5 |  | 2.0 | 3.9 | 3.0 |  |
|  | 35-44 | 404 | 20.7 |  | 457 | 21.7 |  | 861 | 21.2 |  | 11.1 | 18.6 | 15.1 |  | 5.9 | 12.5 | 9.4 |  |
|  | 45-54 | 404 | 20.7 |  | 449 | 21.3 |  | 853 | 21.1 |  | 14.4 | 27.6 | 21.3 |  | 9.9 | 21.4 | 15.9 |  |
|  | 55-64 | 310 | 15.9 |  | 364 | 17.2 |  | 674 | 16.6 |  | 21.6 | 37.4 | 30.1 |  | 12.3 | 28.6 | 21.1 |  |
|  | 65-74 | 187 | 9.7 |  | 179 | 8.5 |  | 366 | 9 |  | 34.8 | 52.5 | 43.4 |  | 21.4 | 29.1 | 25.1 |  |
|  | 75-84 | 78 | 4.1 |  | 93 | 4.4 |  | 171 | 4.2 |  | 39.7 | 52.7 | 46.8 |  | 29.5 | 41.9 | 36.3 |  |
|  | ≥85 | 22 | 1.1 |  | 18 | 0.9 |  | 40 | 1 |  | 31.8 | 61.1 | 45.0 |  | 31.8 | 50.0 | 40.0 |  |
|  | Total | 1948 | 100 |  | 2108 | 100 |  | 4056 | 100 |  | 15.0 | 25.6 | 20.5 |  | 9.4 | 17.8 | 13.8 |  |
|  | Standardized Rate |  |  |  |  |  |  |  |  |  | 11.5* | 20.2 | 15.7 |  | 7.0* | 13.5 | 10.2 |  |

To be continued (S1Table. 2-2）

|  | Age |  | Neck (％) | | |  | Lumbar (％) | | |  | Shoulder (％) | | |  | Elbow (％) | | |  | Foot (％) | | |
| --- | --- | --- | --- | --- | --- | --- | --- | --- | --- | --- | --- | --- | --- | --- | --- | --- | --- | --- | --- | --- | --- |
|  | （yrs） |  | M | F | Total |  | M | F | Total |  | M | F | Total |  | M | F | Total |  | M | F | Total |
| NE | 16-24 |  | 1.5 | 2.9 | 2.2 |  | 1.5 | 1.5 | 1.5 |  | 0.0 | 0.7 | 0.4 |  | 0.7 | 0.0 | 0.4 |  | 0.0 | 0.0 | 0.0 |
|  | 25-34 |  | 3.6 | 7.1 | 5.4 |  | 3.1 | 3.6 | 3.3 |  | 1.0 | 3.1 | 2.1 |  | 1.5 | 1.5 | 1.5 |  | 0.0 | 0.0 | 0.0 |
|  | 35-44 |  | 5.1 | 11.3 | 8.3 |  | 3.7 | 7.1 | 5.5 |  | 3.2 | 3.3 | 3.3 |  | 0.9 | 0.8 | 0.9 |  | 1.4 | 0.8 | 1.1 |
|  | 45-54 |  | 6.8 | 14.9 | 11.2 |  | 5.5 | 13.7 | 10.0 |  | 1.8 | 9.2 | 5.8 |  | 1.8 | 6.9 | 4.6 |  | 2.3 | 3.4 | 2.9 |
|  | 55-64 |  | 14.8 | 11.2 | 12.9 |  | 7.7 | 16.5 | 12.4 |  | 7.7 | 12.9 | 10.5 |  | 2.6 | 8.0 | 5.5 |  | 5.6 | 7.1 | 6.4 |
|  | 65-74 |  | 9.5 | 15.9 | 12.1 |  | 8.6 | 15.9 | 11.6 |  | 12.1 | 12.2 | 12.1 |  | 1.7 | 6.1 | 3.5 |  | 5.2 | 3.7 | 4.5 |
|  | 75-84 |  | 17.9 | 15.4 | 16.5 |  | 12.8 | 15.4 | 14.3 |  | 7.7 | 13.5 | 11.0 |  | 0.0 | 3.8 | 2.2 |  | 10.3 | 3.8 | 6.6 |
|  | ≥85 |  | 7.7 | 7.7 | 7.7 |  | 15.4 | 30.8 | 23.1 |  | 7.7 | 15.4 | 11.5 |  | 0.0 | 0.0 | 0.0 |  | 15.4 | 0.0 | 7.7 |
|  | Total |  | 7.3 | 10.9 | 9.2 |  | 5.3 | 10.3 | 7.9 |  | 4.1 | 7.2 | 5.7 |  | 1.5 | 4.0 | 2.8 |  | 2.7 | 2.7 | 2.7 |
|  | Standardized Rate |  | 5.8*^$^ | 9.6^$^ | 7.6^$^ |  | 4.3*^$^ | 8.0^$^ | 6.1^$^ |  | 2.9* | 5.5^$^ | 4.2^$^ |  | 1.3^&^ | 2.9^$^ | 2.1^$^ |  | 1.8 | 1.7 | 1.7 |
| E | 16-24 |  | 0.0 | 0.0 | 0.0 |  | 0.0 | 1.0 | 0.5 |  | 0.0 | 1.0 | 0.5 |  | 0.0 | 0.0 | 0.0 |  | 0.0 | 0.0 | 0.0 |
|  | 25-34 |  | 1.0 | 0.9 | 0.9 |  | 0.0 | 1.8 | 0.9 |  | 0.0 | 0.0 | 0.0 |  | 0.0 | 0.0 | 0.0 |  | 0.0 | 0.9 | 0.5 |
|  | 35-44 |  | 2.1 | 3.7 | 3.0 |  | 0.5 | 3.2 | 2.0 |  | 2.7 | 0.9 | 1.7 |  | 0.0 | 0.5 | 0.2 |  | 2.1 | 0.0 | 1.0 |
|  | 45-54 |  | 1.6 | 3.7 | 2.7 |  | 0.5 | 3.2 | 1.9 |  | 0.0 | 1.6 | 0.8 |  | 0.0 | 1.1 | 0.5 |  | 2.7 | 1.1 | 1.9 |
|  | 55-64 |  | 2.6 | 11.4 | 7.5 |  | 6.1 | 7.9 | 7.1 |  | 4.4 | 2.9 | 3.5 |  | 0.9 | 0.7 | 0.8 |  | 11.4 | 4.3 | 7.5 |
|  | 65-74 |  | 8.1 | 10.3 | 9.5 |  | 7.1 | 6.2 | 6.5 |  | 7.0 | 6.2 | 6.5 |  | 1.4 | 0.0 | 0.6 |  | 9.9 | 3.1 | 6.0 |
|  | 75-84 |  | 5.1 | 10.0 | 7.5 |  | 7.7 | 10.0 | 8.8 |  | 5.1 | 12.2 | 8.8 |  | 0.0 | 2.4 | 1.3 |  | 12.8 | 2.4 | 7.5 |
|  | ≥85 |  | 11.1 | 20.0 | 14.3 |  | 11.1 | 40.0 | 21.9 |  | 0.0 | 0.0 | 0.0 |  | 11.1 | 0.0 | 7.1 |  | 11.1 | 0.0 | 7.1 |
|  | Total |  | 2.4 | 5.2 | 3.9 |  | 2.2 | 4.3 | 3.3 |  | 2.1 | 2.3 | 2.2 |  | 0.4 | 0.6 | 0.5 |  | 4.3 | 1.4 | 2.8 |
|  | Standardized Rate |  | 1.8^#^ | 3.7 | 2.8 |  | 1.4* | 3.5 | 2.4 |  | 1.5 | 1.7 | 1.6 |  | 0.2 | 0.4 | 0.3 |  | 2.9^#^ | 1.1 | 2.0 |
| Total | 16-24 |  | 0.8 | 1.7 | 1.2 |  | 0.8 | 1.3 | 1.0 |  | 0.0 | 0.8 | 0.4 |  | 0.4 | 0.0 | 0.2 |  | 0.0 | 0.0 | 0.0 |
|  | 25-34 |  | 2.7 | 4.9 | 3.8 |  | 2.0 | 2.9 | 2.5 |  | 0.7 | 1.9 | 1.3 |  | 1.0 | 1.0 | 1.0 |  | 0.0 | 0.3 | 0.2 |
|  | 35-44 |  | 3.7 | 7.7 | 5.8 |  | 2.2 | 5.3 | 3.8 |  | 3.0 | 2.2 | 2.6 |  | 0.5 | 0.6 | 0.6 |  | 1.7 | 0.4 | 1.0 |
|  | 45-54 |  | 4.5 | 10.2 | 7.6 |  | 3.2 | 9.4 | 6.4 |  | 1.0 | 6.0 | 3.6 |  | 1.0 | 4.5 | 2.8 |  | 2.5 | 2.5 | 2.5 |
|  | 55-64 |  | 10.3 | 11.3 | 10.8 |  | 7.1 | 13.2 | 10.4 |  | 6.5 | 9.1 | 7.9 |  | 1.9 | 5.2 | 3.7 |  | 7.7 | 6.0 | 6.8 |
|  | 65-74 |  | 9.1 | 12.8 | 10.9 |  | 8.0 | 10.6 | 9.3 |  | 10.1 | 8.9 | 9.6 |  | 1.6 | 2.8 | 2.2 |  | 7.0 | 3.4 | 5.2 |
|  | 75-84 |  | 11.5 | 12.9 | 12.3 |  | 10.3 | 12.9 | 11.7 |  | 6.4 | 12.9 | 9.9 |  | 0.0 | 3.2 | 1.8 |  | 11.5 | 3.2 | 7.0 |
|  | ≥85 |  | 9.1 | 11.1 | 10.0 |  | 13.6 | 33.3 | 22.5 |  | 4.5 | 11.1 | 7.5 |  | 4.5 | 0.0 | 2.5 |  | 13.6 | 0.0 | 7.5 |
|  | Total |  | 5.4 | 8.5 | 7.0 |  | 4.0 | 7.7 | 5.9 |  | 3.2 | 5.1 | 4.2 |  | 1.0 | 2.5 | 1.8 |  | 3.3 | 2.2 | 2.7 |
|  | Standardized Rate |  | 4.2* | 7.1 | 5.6 |  | 3.1* | 6.0 | 4.5 |  | 2.4^#^ | 3.9 | 3.1 |  | 0.9 | 1.8 | 1.4 |  | 2.2 | 1.4 | 1.8 |

Comparison with female*=*P*<0.01， #= *P*<0.05； Comparison with building s with elevators^$^ = *P*<0.01， ^&^= *P*<0.05
