# Supplementary material for: Changes in the Prevalence of Rheumatic Diseases in Shantou, China, in the Past Three Decades: A COPCORD Study
Source: PLoS One. 2015 Sep 25;10(9):e0138492. doi: 10.1371/journal.pone.0138492 (PMC4583180; doi:10.1371/journal.pone.0138492)
Supplement: S2 Table — (DOC) [file pone.0138492.s002.doc]

**S2Table.** Prevalence Rates and Occupational Distribution of Common Rheumatic Diseases in Shantou, 2012（2-1）

|  |  |  |  | Student |  |  |  |  | Teacher |  |  |  |  | Individual |  |  |  |  |
| --- | --- | --- | --- | --- | --- | --- | --- | --- | --- | --- | --- | --- | --- | --- | --- | --- | --- | --- |
|  |  | N | % | Sd. R | 95%CI |  | N | % | Sd. R | 95%CI |  | N | % | Sd. R | 95%CI |  |  |  |
| Total | total | 291 | 7.2 |  |  |  | 325 | 8 |  |  |  | 509 | 12.5 |  |  |  |  |  |
|  | NE | 206 | 8.8 |  |  |  | 211 | 9.1 |  |  |  | 215 | 9.2 |  |  |  |  |  |
|  | E | 85 | 5 |  |  |  | 114 | 6.6 |  |  |  | 294 | 17.1 |  |  |  |  |  |
| NE | RP | 8 | 3.88 | 0.73 | 0, 1.89 |  | 59 | 27.96 | 21.53 | 15.98, 27.08 |  | 37 | 17.21 | 14.28 | 9.60, 18.96 |  |  |  |
|  | KP | 6 | 2.91 | 0.55 | 0, 1.56 |  | 36 | 17.06 | 12.43 | 7.98, 10.88 |  | 32 | 14.88 | 10.90 | 6.73, 15.07 |  |  |  |
|  | KOA | 1 | 0.49 | 0.09 | 0, 0.50 |  | 27 | 12.8 | 9.61 | 5.63, 13.69 |  | 9 | 4.19 | 2.78 | 0.58, 4.98 |  |  |  |
|  | Gout | 0 | 0 | 0 |  |  | 5 | 2.37 | 1.82 | 0.02, 3.62 |  | 6 | 2.79 | 1.85 | 0.05, 3.65 |  |  |  |
|  | RA | 0 |  |  |  |  | 0 |  |  |  |  | 1 | 0.5 |  |  |  |  |  |
|  | AS | 0 |  |  |  |  | 1 | 0.50 |  |  |  | 0 |  |  |  |  |  |  |
|  | FM |  |  |  |  |  |  |  |  |  |  | 0 |  |  |  |  |  |  |
| E | RP | 3 | 3.53 | 0.67 | 0, 2.40 |  | 17 | 14.91 | 9.58 | 4.18, 14.98 |  | 28 | 9.52 | 10.91 | 7.35, 14.47 |  |  |  |
|  | KP | 1 | 1.18 | 0.23 | 0, 1.25 |  | 14 | 12.28 | 8.36 | 3.28, 13.44 |  | 14 | 4.76 | 5.35 | 2.78, 7.92 |  |  |  |
|  | KOA | 0 | 0 | 0 |  |  | 13 | 11.4 | 7.03 | 2.34,11.72 |  | 11 | 3.74 | 4.06 | 1.80, 6.32 |  |  |  |
|  | Gout | 0 | 0 | 0 |  |  | 1 | 0.88 | 0.32 | 0, 1.36 |  | 6 | 2.04 | 1.87 | 0.32, 3.42 |  |  |  |
|  | RA | 0 |  |  |  |  | 0 |  |  |  |  | 1 | 0.34 |  |  |  |  |  |
|  | AS | 0 |  |  |  |  | 1 | 0.90 |  |  |  | 1 | 0.34 |  |  |  |  |  |
|  | FM |  |  |  |  |  | 1 | 0.88 |  |  |  | 0 |  |  |  |  |  |  |
| Total | RP | 11 | 3.78 | 0.71 | 0, 1.67 |  | 76 | 23.38 | 17.46 | 13.33, 21.59 |  | 65 | 12.77 | 12.12 | 9.28, 14.96 |  |  |  |
|  | KP | 7 | 2.41 | 0.46 | 0, 1.24 |  | 50 | 15.38 | 11.35 | 7.90, 14.80 |  | 46 | 9.04 | 8.25 | 5.86, 10.64 |  |  |  |
|  | KOA | 1 | 0.34 | 0.07 | 0, 0.37 |  | 40 | 12.31 | 8.83 | 5.75, 11.91 |  | 20 | 3.93 | 3.44 | 1.86, 5.02 |  |  |  |
|  | Gout | 0 | 0 | 0 |  |  | 6 | 1.85 | 1.44 | 0.14, 2.74 |  | 12 | 2.36 | 1.97 | 0.76, 3.18 |  |  |  |
|  | RA | 0 |  |  |  |  | 0 |  |  |  |  | 2 | 0.39 |  |  |  |  |  |
|  | AS | 0 |  |  |  |  | 2 | 0.62 |  |  |  | 1 | 0.20 |  |  |  |  |  |
|  | FM | 0 |  |  |  |  | 1 | 0.31 |  |  |  | 0 |  |  |  |  |  |  |

To be continued (S2 Table. 2-2）

|  |  |  |  |  |  | Blue-collar |  |  |  |  | White-collar |  |  |  |  | Total |  |
| --- | --- | --- | --- | --- | --- | --- | --- | --- | --- | --- | --- | --- | --- | --- | --- | --- | --- |
|  |  |  |  | N | % | Sd. R | 95%CI |  | N | % | Sd. R | 95%CI |  | N | % | Sd. R | 95%CI |
| Total | total |  |  | 1263 | 31.1 |  |  |  | 1668 | 40.9 |  |  |  | 4056 | 100 |  |  |
|  | NE |  |  | 873 | 37.4 |  |  |  | 832 | 35.2 |  |  |  | 2337 | 57.6 |  |  |
|  | E |  |  | 390 | 22.7 |  |  |  | 836 | 48.5 |  |  |  | 1719 | 42.4 |  |  |
| NE | RP |  |  | 284 | 32.53 | 19.83 | 17.19, 22.47 |  | 177 | 21.27 | 16.26 | 13.75, 18.77 |  | 565 | 24.18 | 18.98 | 17.39, 20.57 |
|  | KP |  |  | 180 | 20.62 | 12.49 | 10.30, 14.68 |  | 109 | 13.10 | 9.18 | 7.22, 11.14 |  | 363 | 15.53 | 11.54 | 10.24, 12.84 |
|  | KOA |  |  | 151 | 17.30 | 10.83 | 8.77, 12.89 |  | 68 | 8.17 | 5.45 | 3.91, 6.99 |  | 256 | 10.95 | 7.76 | 6.68,8.84 |
|  | Gout |  |  | 13 | 1.49 | 0.99 | 0.33, 1.65 |  | 9 | 1.08 | 0.93 | 0.28, 1.58 |  | 33 | 1.41 | 1.12 | 0.69, 1.55 |
|  | RA |  |  | 5 | 0.57 |  |  |  | 4 | 0.48 |  |  |  | 10 | 0.43 | 0.35 | 0.11, 0.59 |
|  | AS |  |  | 2 | 0.23 |  |  |  | 4 | 0.48 |  |  |  | 7 | 0.3 | 0.31 | 0.08, 0.54 |
|  | FM |  |  | 2 | 0.23 |  |  |  | 2 | 0.24 |  |  |  | 4 | 0.17 | 0.10 | 0, 0.23 |
| E | RP |  |  | 87 | 22.31 | 11.44 | 8.28, 14.60 |  | 131 | 15.67 | 10.23 | 8.18, 12.28 |  | 266 | 15.47 | 11.35 | 9.85, 12.85 |
|  | KP |  |  | 72 | 18.46 | 12.46 | 9.18, 15.74 |  | 96 | 11.48 | 9.21 | 7.25, 11.17 |  | 197 | 11.46 | 8.9 | 7.55, 10.25 |
|  | KOA |  |  | 55 | 14.10 | 7.98 | 5.29, 10.67 |  | 86 | 10.29 | 6.59 | 4.91, 8.27 |  | 165 | 9.6 | 6.49 | 5.33, 7.65 |
|  | Gout |  |  | 9 | 2.31 | 0.81 | 0, 1.70 |  | 7 | 0.84 | 0.54 | 0.04, 1.04 |  | 23 | 1.34 | 0.9 | 0.45, 1.35 |
|  | RA |  |  | 3 | 0.77 |  |  |  | 5 | 0.60 |  |  |  | 9 | 0.52 | 0.35 | 0.07, 0.63 |
|  | AS |  |  | 1 | 0.26 |  |  |  | 2 | 0.24 |  |  |  | 5 | 0.12 | 0.31 | 0.05, 0.57 |
|  | FM |  |  | 0 | 0 |  |  |  | 0 | 0 |  |  |  | 1 | 0.06 | 0.03 | 0, 0.17 |
| Total | RP |  |  | 371 | 29.37 | 17.24 | 15.16, 19.32 |  | 308 | 18.47 | 13.64 | 11.99, 15.29 |  | 831 | 20.49 | 15.77 | 14.69, 16.89 |
|  | KP |  |  | 252 | 19.95 | 12.47 | 10.65, 14.29 |  | 205 | 12.29 | 9.21 | 7.82, 10.60 |  | 560 | 13.81 | 10.4 | 9.46, 11.34 |
|  | KOA |  |  | 206 | 16.31 | 9.83 | 8.19, 11.47 |  | 154 | 9.23 | 6.07 | 4.92, 7.22 |  | 421 | 10.38 | 7.29 | 6.49, 8.09 |
|  | Gout |  |  | 22 | 1.74 | 1.01 | 0.46, 1.56 |  | 16 | 0.96 | 0.79 | 0.37, 1.21 |  | 56 | 1.38 | 1.04 | 0.73, 1.35 |
|  | RA |  |  | 8 | 0.63 |  |  |  | 9 | 0.54 |  |  |  | 19 | 0.47 | 0.35 | 0.17, 0.53 |
|  | AS |  |  | 3 | 0.24 |  |  |  | 6 | 0.36 |  |  |  | 12 | 0.3 | 0.31 | 0.14, 0.48 |
|  | FM |  |  | 2 | 0.16 |  |  |  | 2 | 0.12 |  |  |  | 5 | 0.12 | 0.07 | 0, 0.15 |

Abbreviations: AS, ankylosing spondylitis; Blue-collar, blue collar worker; FM, fibromyalgia; Individual, individual industrialists and businessmen; KP, knee pain; KOA, knee osteoarthritis; RA, rheumatoid arthritis; Sd. R, standardized rate; White-collar, white collar worker.

* N-E=Building without elevators, E= Building with elevators;
