# Supplementary material for: Changes in the Prevalence of Rheumatic Diseases in Shantou, China, in the Past Three Decades: A COPCORD Study
Source: PLoS One. 2015 Sep 25;10(9):e0138492. doi: 10.1371/journal.pone.0138492 (PMC4583180; doi:10.1371/journal.pone.0138492)
Supplement: S3 Table — (DOC) [file pone.0138492.s003.doc]

**S3Table.** Age, Sex, and Housing Distribution of Common Rheumatic Diseases in Shantou, 2012

| Age | M | F | Total |  | KOA Prev. (％) | | |  | Gout Prev. (％) | | |  | RA Prev. (％) | | |  | AS Prev.(％) | | |  | FM Prev. (％) | | |
| --- | --- | --- | --- | --- | --- | --- | --- | --- | --- | --- | --- | --- | --- | --- | --- | --- | --- | --- | --- | --- | --- | --- | --- |
| （yrs） | n | n | n |  | M | F | Total |  | M | F | Total |  | M | F | Totl |  | M | F | Total |  | M | F | Total |
| N-E |  |  |  |  |  |  |  |  |  |  |  |  |  |  |  |  |  |  |  |  |  |  |  |
| 16-24 | 136 | 138 | 274 |  | 0.73 | 1.45 | 1.09 |  | 0 | 0 | 0 |  | 0 | 0 | 0 |  | 0.73 | 0 | 0.36 |  | 0 | 0 | 0 |
| 25-34 | 194 | 196 | 390 |  | 0.52 | 3.57 | 2.05 |  | 1.03 | 0.51 | 0.77 |  | 0 | 0.51 | 0.26 |  | 0.52 | 0 | 0.26 |  | 0 | 0 | 0 |
| 35-44 | 217 | 239 | 456 |  | 1.84 | 4.6 | 3.29 |  | 2.30 | 0 | 1.10 |  | 0.46 | 0.42 | 0.44 |  | 0.92 | 0 | 0.44 |  | 0 | 0 | 0 |
| 45-54 | 220 | 262 | 482 |  | 7.73 | 19.85 | 14.32 |  | 2.27 | 0.38 | 1.24 |  | 0.45 | 0.76 | 0.62 |  | 0 | 0.38 | 0.21 |  | 0 | 0.38 | 0.21 |
| 55-64 | 196 | 224 | 420 |  | 11.73 | 27.23 | 20 |  | 4.59 | 0.89 | 2.62 |  | 0.51 | 0.89 | 0.71 |  | 0.51 | 0.45 | 0.48 |  | 0 | 0.89 | 0.48 |
| 65-74 | 116 | 82 | 198 |  | 13.79 | 30.49 | 20.7 |  | 5.17 | 0 | 3.03 |  | 0 | 1.22 | 0.51 |  | 0 | 0 | 0 |  | 0 | 0 | 0 |
| 75-84 | 39 | 52 | 91 |  | 28.2 | 25 | 26.37 |  | 5.13 | 0 | 2.20 |  | 0 | 0 | 0 |  | 0 | 0 | 0 |  | 0 | 1.92 | 1.10 |
| ≥85 | 13 | 13 | 26 |  | 38.46 | 53.55 | 46.15 |  | 0 | 0 | 0 |  | 0 | 0 | 0 |  | 0 | 0 | 0 |  | 0 | 0 | 0 |
| Total(N) | 1131 | 1206 | 2337 |  | 78 | 178 | 256 |  | 29 | 4 | 33 |  | 3 | 7 | 10 |  | 5 | 2 | 7 |  | 0 | 4 | 4 |
| Prev.％ |  |  |  |  | 6.90 | 14.76 | 11.00 |  | 2.56 | 0.33 | 1.41 |  | 0.27 | 0.58 | 0.43 |  | 0.44 | 0.17 | 0.30 |  | 0 | 0.33 | 0.17 |
| Sd. R％ |  |  |  |  | 4.60 | 10.8 | 7.64 |  | 1.98 | 0.27 | 1.15 |  | 0.22 | 0.50 | 0.35 |  | 0.51 | 0.10 | 0.31 |  | 0 | 0.20 | 0.10 |
|  |  |  |  |  |  |  |  |  |  |  |  |  |  |  |  |  |  |  |  |  |  |  |  |
| E |  |  |  |  |  |  |  |  |  |  |  |  |  |  |  |  |  |  |  |  |  |  |  |
| 16-24 | 112 | 101 | 213 |  | 0 | 0 | 0 |  | 0 | 0 | 0 |  | 0 | 0 | 0 |  | 0 | 0 | 0 |  | 0 | 0 | 0 |
| 25-34 | 101 | 113 | 214 |  | 0 | 0.88 | 0.47 |  | 0 | 0 | 0 |  | 0 | 0 | 0 |  | 0.99 | 0 | 0.47 |  | 0 | 0 | 0 |
| 35-44 | 187 | 218 | 405 |  | 1.05 | 5.05 | 3.20 |  | 1.60 | 0 | 0.74 |  | 0 | 0.46 | 0.25 |  | 0.53 | 0 | 0.25 |  | 0 | 0 | 0 |
| 45-54 | 184 | 187 | 371 |  | 2.71 | 13.36 | 8.09 |  | 1.09 | 0 | 0.54 |  | 1.09 | 0.53 | 0.81 |  | 0.54 | 0.53 | 0.54 |  | 0 | 0 | 0 |
| 55-64 | 114 | 140 | 254 |  | 10.53 | 24.29 | 18.11 |  | 3.51 | 1.43 | 2.36 |  | 0 | 2.14 | 1.18 |  | 0.88 | 0 | 0.39 |  | 0 | 0.71 | 0.39 |
| 65-74 | 71 | 97 | 168 |  | 18.31 | 36.08 | 28.57 |  | 11.27 | 2.08 | 5.95 |  | 1.41 | 1.03 | 1.19 |  | 0 | 0 | 0 |  | 0 | 0 | 0 |
| 75-84 | 39 | 41 | 80 |  | 17.95 | 41.46 | 30.00 |  | 2.56 | 0 | 1.25 |  | 0 | 0 | 0 |  | 0 | 0 | 0 |  | 0 | 0 | 0 |
| ≥85 | 9 | 5 | 14 |  | 11.11 | 40.00 | 21.43 |  | 11.11 | 0 | 7.14 |  | 0 | 0 | 0 |  | 0 | 0 | 0 |  | 0 | 0 | 0 |
| Total | 817 | 902 | 1719 |  | 40 | 125 | 165 |  | 19 | 4 | 23 |  | 3 | 6 | 9 |  | 4 | 1 | 5 |  | 0 | 1 | 1 |
| Prev.％ |  |  |  |  | 4.90 | 13.86 | 9.60 |  | 2.33 | 0.44 | 1.34 |  | 0.37 | 0.67 | 0.52 |  | 0.49 | 0.11 | 0.29 |  | 0 | 0.11 | 0.06 |
| Sd. R％ |  |  |  |  | 3.22 | 9.41 | 6.26 |  | 1.63 | 0.27 | 0.96 |  | 0.26 | 0.44 | 0.35 |  | 0.54 | 0.08 | 0.31 |  | 0 | 0.07 | 0.03 |
|  |  |  |  |  |  |  |  |  |  |  |  |  |  |  |  |  |  |  |  |  |  |  |  |
| Total |  |  |  |  |  |  |  |  |  |  |  |  |  |  |  |  |  |  |  |  |  |  |  |
| 16-24 | 248 | 239 | 487 |  | 0.40 | 0.84 | 0.62 |  | 0 | 0 | 0 |  | 0 | 0 | 0 |  | 0.4 | 0 | 0.2 |  | 0 | 0 | 0 |
| 25-34 | 295 | 309 | 604 |  | 0.34 | 2.59 | 1.49 |  | 0.68 | 0.32 | 0.50 |  | 0 | 0.32 | 0.16 |  | 0.68 | 0 | 0.33 |  | 0 | 0 | 0 |
| 35-44 | 404 | 457 | 861 |  | 1.49 | 4.81 | 3.25 |  | 1.98 | 0 | 0.93 |  | 0.25 | 0.44 | 0.35 |  | 0.74 | 0 | 0.35 |  | 0 | 0 | 0 |
| 45-54 | 404 | 449 | 853 |  | 5.45 | 17.15 | 11.61 |  | 1.73 | 0.22 | 0.94 |  | 0.74 | 0.67 | 0.7 |  | 0.25 | 0.45 | 0.35 |  | 0 | 0.22 | 0.12 |
| 55-64 | 310 | 364 | 674 |  | 11.29 | 26.10 | 19.29 |  | 4.19 | 1.10 | 2.52 |  | 0.32 | 1.37 | 0.89 |  | 0.65 | 0.27 | 0.45 |  | 0 | 0.82 | 0.45 |
| 65-74 | 187 | 179 | 366 |  | 15.51 | 33.52 | 24.30 |  | 7.49 | 1.12 | 4.37 |  | 0.53 | 1.11 | 0.82 |  | 0 | 0 | 0 |  | 0 | 0 | 0 |
| 75-84 | 78 | 93 | 171 |  | 23.08 | 32.26 | 28.07 |  | 3.85 | 0 | 1.75 |  | 0 | 0 | 0 |  | 0 | 0 | 0 |  | 0 | 1.08 | 0.58 |
| ≥85 | 22 | 18 | 40 |  | 27.27 | 50.00 | 37.50 |  | 4.55 | 0 | 2.50 |  | 0 | 0 | 0 |  | 0 | 0 | 0 |  | 0 | 0 | 0 |
| Total | 1948 | 2108 | 4056 |  | 118 | 303 | 421 |  | 48 | 8 | 56 |  | 6 | 13 | 19 |  | 9 | 3 | 12 |  | 0 | 5 | 5 |
| Prev.％ |  |  |  |  | 6.06 | 14.37 | 10.38 |  | 2.46 | 0.38 | 1.38 |  | 0.31 | 0.62 | 0.47 |  | 0.46 | 0.14 | 0.30 |  | 0 | 0.24 | 0.12 |
| Sd. R％ |  |  |  |  | 3.97 | 10.35 | 7.10 |  | 1.82 | 0.29 | 1.08 |  | 0.23 | 0.48 | 0.35 |  | 0.51 | 0.10 | 0.31 |  | 0 | 0.14 | 0.07 |

Abbreviations: AS, ankylosing spondylitis; FM, fibromyalgia; KP, knee pain; KOA, knee osteoarthritis; Prev., prevalence; RA, rheumatoid arthritis; Sd. R, standardized rate; * N-E=Building without elevators, E= Building with elevators
